# Effects of a multi-modal static and dynamic balance and hand function training program on multi-dimensional balance ability, hand function, and life-space mobility in rural older adults: protocol for a randomized controlled trial

**DOI:** 10.1186/s13102-025-01214-2

**Published:** 2025-07-02

**Authors:** Xinyu Yang, Shasha Li, Xiangdong Xu, Guojing Guo, Xiaofang Song, Yashuang Sun, Mengxue Shi, Canqi Ren

**Affiliations:** 1 Department of Nursing, College of Medical Science, Huzhou University, 759 Second Ring Road, Huzhou City, Zhejiang Province 313000 China; 2 Center for Disease Control and Prevention, Huzhou City Nanxun District, 660 lin Middle Road, Nanxun Town, Nanxun District, Huzhou City, Zhejiang Province 313009 China

**Keywords:** Health intervention, Exercise program evaluation, Aging-related mobility improvement, Balance ability

## Abstract

**Background:**

Rural older adults face an increasing number of health challenges. While balance training is beneficial, it is often included as part of broader exercise programs. However, comprehensive studies specifically combining balance training with hand function training for rural older adults remain scarce.

**Objective:**

This study aims to investigate whether a combined balance and hand training program can provide programmatic references and empirical evidence for improving functional outcomes in rural older adults.

**Methods:**

A single-blind, four-group parallel, multicenter randomized controlled trial was used. Four rural communities in Huzhou, Zhejiang Province, were included in the study, with an estimated total sample size of 132 individuals. During the 16-week intervention period, the comprehensive exercise program consisted of two balance training programs and one hand training session provided by a multidisciplinary team including physiotherapists, sports professionals, nurses and community health workers. The four rural communities were randomly divided into four experimental groups in the ratio of 1:1:1:1 (a) static balance training combined with hand training; (b) dynamic balance training combined with a hand training program; (c) multi-modal static-dynamic balance and hand function training (M-mBHF) program; and (d) a Daily activity group. The multidimensional balance ability, hand function, and life-space mobility of the four parallel groups will be assessed at baseline (T0), 8 weeks of intervention (T1), 16 weeks of intervention (T2), and at 24 (T3) and 32 weeks (T4) follow-up.

**Conclusion:**

This study protocol proposes a M-mBHF program hypothesized to improve multi-dimensional balance ability, hand function, and life-space mobility in rural older adults. The anticipated outcomes aim to provide empirical evidence for future interventions targeting this population.

**Trial registration:**

Registered in the Chinese Clinical Trial Registry on 02/17/2025 (ChiCTR2500097310).

**Supplementary Information:**

The online version contains supplementary material available at 10.1186/s13102-025-01214-2.

## Introduction

With the evolution of the global population structure, it is projected that the number of older adults aged 60 and above will double to 2.1 billion by 2050. In 2024, the World Health Organization (WHO) highlighted that environmental factors have significantly impacted the health of older adults, particularly in rural areas of developing countries, where environmental limitations and rapidly rising living costs exacerbate challenges [1]. As of 2024, China’s older adults aged 60 and above have reached 310 million, accounting for 22.0% of the total population. Notably, rural areas face a more pronounced aging issue, with 23.81% of the population being older adults significantly higher than in urban areas [2]. 

Rural older adults often encounter more severe issues regarding access to health services and resources [3], leading to a higher likelihood of physical decline [4]. Long-term agricultural labor can accelerate the degradation of skeletal muscle function, impair balance, and increase fall risks due to complex terrains and limited health awareness. Data indicate that the fall mortality rate among rural older adults (9.95/100,000) is notably higher than in urban areas (7.18/100,000).[5] Studies show that older adults exhibit slower reactive abilities in fingers and arms, contributing to reduced grip strength compared to younger adults [6]. Additionally, long-term manual labor results in hand muscle strain, rough skin, and decreased tactile sensitivity, further compromising grip stability. Therefore, focusing on balance and hand function is crucial for promoting healthy aging in rural older adults.

Life-space mobility (LSM) is a comprehensive and dynamic measure that reflects the interaction between individual functions, environment, and society [7]. Compared to laboratory-based assessments, LSM provides a more accurate reflection of real-life physical activities and functions, serving as a strong predictor of adverse outcomes such as falls, hospitalization, and premature death [8]. According to the Webber framework, physical performance, including strength and balance, significantly influences LSM [9]. Kuspinar et al. found that lower walking speed, TUG test scores, and grip strength are associated with reduced LSM [8]. Uchida et al. confirmed that psychological factors, such as fear of falling, depression, and anxiety also impact LSM [10], while poor infrastructure, uneven sidewalks, and noisy traffic in rural areas contribute to mobility disorders, restricting life-space mobility [11]. 

Somatic balance ability encompasses static/dynamic steady-state balance, active balance, and reactive balance [12]. Static balance training involves maintaining independent static postures and low-intensity stability exercises. Ni’s research demonstrated significant improvements in SPPB and One Leg Stand Test scores among community-dwelling older adults after sensory-based static balance training [13]. Granacher et al. reported that an 8-week home-based static gait balance program enhanced active balance and lower limb strength in healthy older adults individuals aged 60-72 [14]. Dynamic balance training aims to improve high-intensity stability and coordination. Hagovska et al. observed significant improvements in walking speed, step length, and body posture control after ten weeks of dynamic balance training [15]. While both static and dynamic training improves balance, their combined effect on multidimensional balance and LSM remains understudied.

Despite WHO recommendations for at least 2.5 h of moderate physical activity per week for adults, rural older adults (≥ 65 years old) still have the highest sedentary time [16]. Rural older adults lacking scientific guidance, often neglect functional balance improvement. Given this context, this study, based on the environmental conditions of rural areas and the characteristics of the older adults’ physical functions (balance ability and hand function) in rural areas, attempts to open a novel M-mBHT program to address potential gaps in current interventions. The objectives of this study are as follows: (1) To construct a scientifically feasible comprehensive intervention plan combining static-dynamic balance training and hand training, promoting the application and dissemination of the intervention plan in rural community older adults; (2) To provide theoretical references for investigating the hypothesized short-term and long-term effects of comprehensive multi-dimensional balance ability, hand function, and life-space mobility for rural older adults.

## Methods

### Study design and ethical consideration

**Fig. 1:**
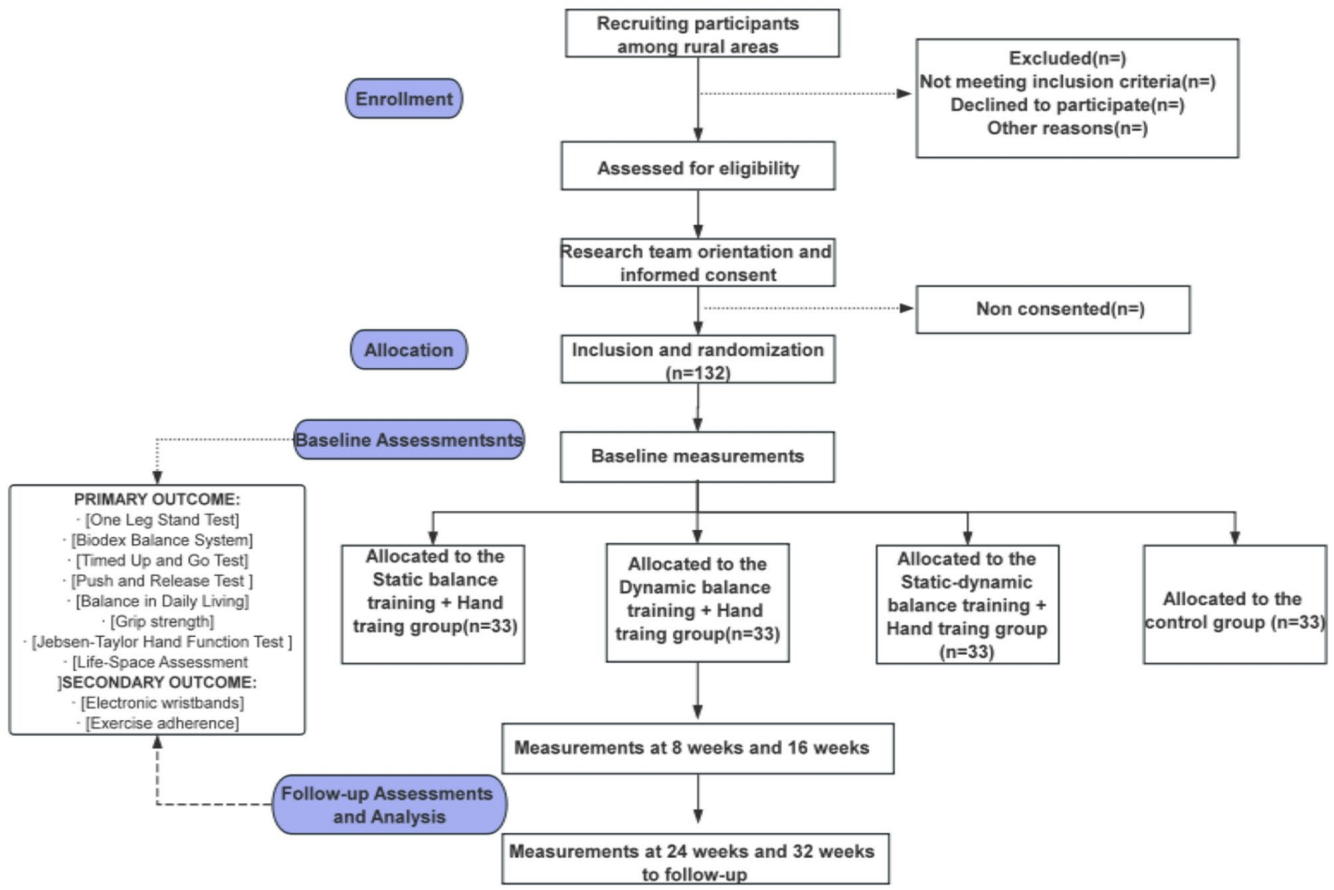
Study design flow chart

A multi-center, four-arm, parallel-group, assessor-blind randomized controlled trial and will take place in four rural communities of Huzhou City, northern Zhejiang, China. The study plan is designed to include three intervention groups: (a) static balance training combined with hand training; (b) dynamic balance training combined with hand training; (c) combined static and dynamic balance training with hand training; and control group (physical and health education).The study process is illustrated in Fig. 1. This study adheres to the CONSORT statement and SPIRIT guidelines [17, 18]. The primary or secondary outcomes were not expected to change during the course of the study. All results were specified in the protocol registered in the Chinese Clinical Trial Register (ChiCTR2500097310). It has been approved by the Medical Ethics Committee of Huzhou University (approval number: 202412-05).

### Participants

Eligible participants for the trial are older adults residing in rural areas. Inclusion Criteria: (1) Age ≥ 60 years; (2) Independent walking ability for ≥ 15 consecutive minutes; (3) No communication barriers; (4) Long-term rural resident (≥ 1 year); (5) Normal or corrected-to-normal visual and hearing acuity (e.g., using glasses/hearing aids); (6) Provided informed consent and willingness to cooperate.Exclusion Criteria: (1) Untreated mental illness; (2) Concurrent participation in related sports training/drug trials; (3) Inability to adhere to the intervention protocol.

### Recruitment and informed consent

Recruitment flyers were distributed by two trained investigators through community centers and local health clinics, and individuals meeting the inclusion criteria were invited to sign informed consent forms. Additionally, local community health workers assisted in referring potential participants to the study. Interested seniors could contact the study investigators, who would explain the research and assess eligibility through telephone or face-to-face interviews.

### Sample size

Based on a previous study evaluating the impact of proprioceptive balance training on posture control in older adults [19], G*Power 3.1 was used for power analysis to determine the required sample size for detecting a statistically significant effect of the intervention [20]. The significance level (α) was set at 0.05, and the statistical power was set at 0.80. To account for an anticipated 10% dropout rate over the 16-week study period, 33 participants will be recruited per group.

### Randomization, allocation concealment, and blinding

Four rural communities in Huzhou, Zhejiang Province, were randomly selected to ensure comparability regarding economic development, cultural practices, and infrastructure. A minimum separation distance of 5 km between communities was maintained to minimize contamination risk. The investigator prepared slips labeled “Intervention groups (a)(b)(c)” and “control group” and placed them in sealed envelopes. Representatives from the four communities randomly selected envelopes and were assigned to the four groups on a 1:1:1:1 ratio. Research assistants received intervention information via sealed envelopes, while outcome evaluators and data analysts remained blinded to the intervention details. Due to the nature of the intervention, blinding participants was not feasible.

### Intervention group

The M-mBHT program was developed by a multidisciplinary team comprising nursing specialists, physiotherapists, psychologists, sociologists, and geriatricians. This team adhered to the ICFSR guidelines to design an integrated training program tailored specifically for older adults in rural communities [21]. The static-dynamic balance training component was derived from balance posture control movements [22], while the hand training component was designed by Ge Shouping, a senior gymnastics coach in Shanghai, using principles of traditional Chinese medicine. The program follows a cyclic training mode, consisting of one week of instruction followed by one week of practice, facilitating steady skill acquisition and internalization by older adults. Training intensity progressively increases through altered sensory inputs and external distractions. The comprehensive program includes four parts: (1) a 5-10-minute warm-up session (including wrist, hip, shoulder, knee, and ankle range of motion exercises); (2) a 15-minute static balance training session (standing on both feet, tandem standing, and one-foot standing); (3) a 25-minute dynamic balance training session (normal gait, narrow gait, overlapping gait, and tandem gait); and (4) a 10-minute hand training session (finger exercises, acupuncture point stimulation, and finger relaxation phases). Detailed information is provided in Table 1. Based on the components of the comprehensive program, the three intervention groups are designated as follows: (a) Static Balance Training with Hand Training, (b) Dynamic Balance Training with Hand Training, and (c) multi-modal static-dynamic balance and hand function training (M-mBHF).

**Table 1:**
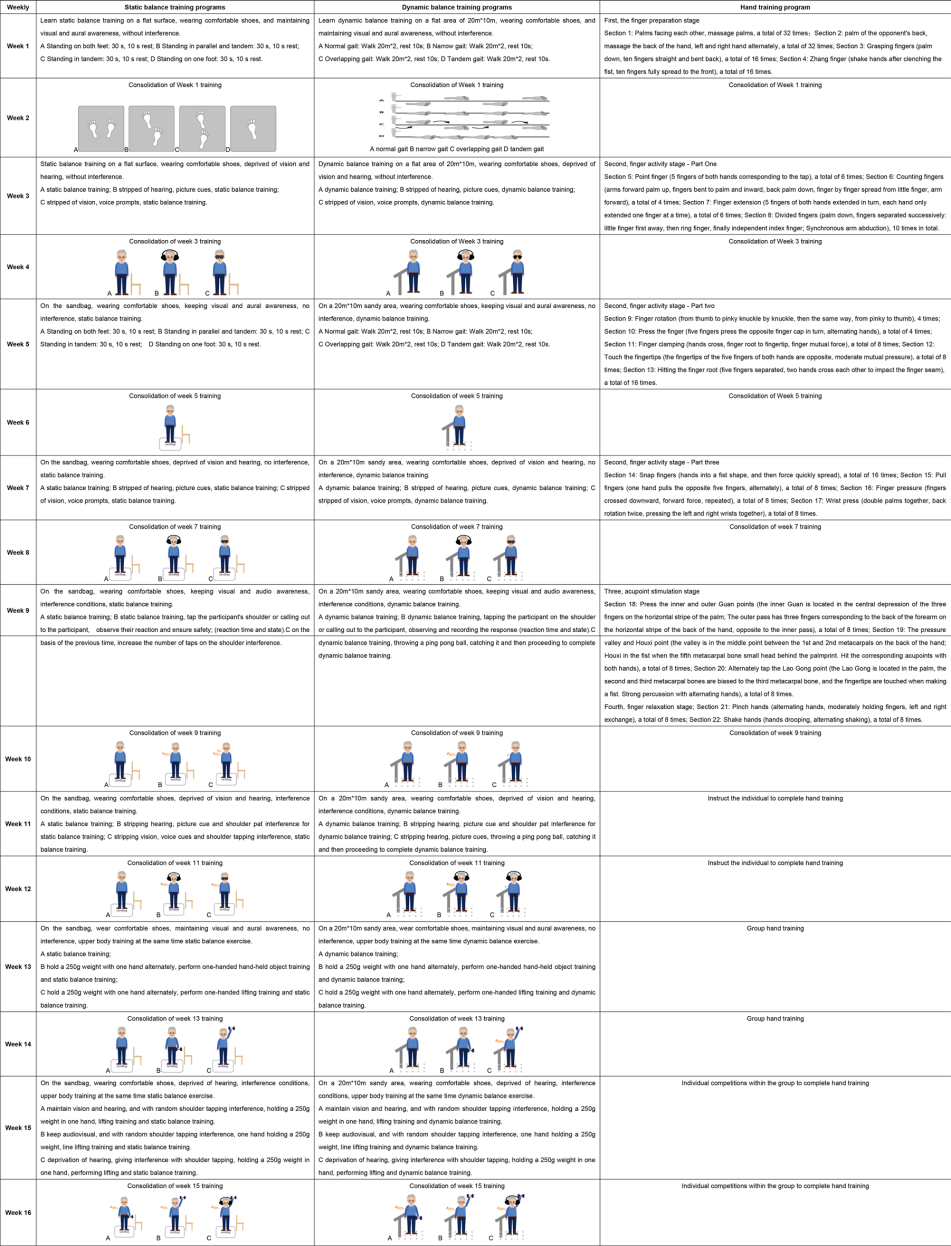
Static balance training dynamic balance training and hand training programs

The intervention team comprised a sports professional, two general practitioners, three nurses, three community health workers, and four volunteer medical graduate students. The intervention program lasted 16 weeks, with sessions held twice weekly: one guided group session and one home-based independent exercise session using the same protocols listed in Table 1. Home exercises were self-administered by participants, but each intervention group was assigned to a dedicated WeChat group for remote supervision. Weekly instructional videos of training content were uploaded to the group chat, and research assistants provided online guidance to address execution errors or safety concerns. Participants self-monitored via WeChat video check-ins or exercise logs, allowing flexible adjustment of exercise intensity based on home environment conditions. Although direct in-person supervision was absent, the research team conducted weekly telephone follow-ups to monitor safety and protocol adherence, each lasting 50–60 min. Follow-up assessments were conducted at 8 and 16 weeks post-intervention.

Participants in all three intervention groups were instructed to maintain their daily lifestyles and avoid seeking any cognitively related information from external sources during the intervention period. They were also asked to report any changes in daily habits, including supplement and drug use, participation in recreational clubs, hospitalizations, and illness conditions. To mitigate potential risks associated with unsupervised exercise, all participants underwent baseline physical assessments and received detailed safety guidelines for home practice, including using stable furniture as support and emergency contact protocols. The multi-disciplinary team (including general practitioners) was accessible for urgent consultations to address health status changes promptly.

### Control group

The control group participated in a weekly 40-minute exercise health education session for 16 weeks. The curriculum covered routine exercises (including cardiovascular, flexibility, and resistance training), warm-up preparations, appropriate exercise duration, frequency, and intensity, exercise precautions, and fall prevention strategies during daily activities. These sessions were led by one health promotion professional and two community health workers. Follow-up assessments were also conducted at 8 and 16 weeks post-intervention.

## Measurement

### Basic information

Basic information collected included age, gender, educational level, marital status, living alone status, monthly income, body mass index (BMI), weekly exercise frequency, and number of falls.

### Primary outcomes

Primary outcome assessments focused on multidimensional balance ability (static, dynamic, proactive, reactive, and daily living balance), hand function (grip strength and manual dexterity), and life-space mobility.

### Static balance ability

Was evaluated using the One Leg Stand Test (OLST). Participants were instructed to lift one leg off the ground (either leg), stand on the remaining leg with eyes open and gaze forward, and maintain this position for as long as possible. Timing began when one foot left the ground. Shorter durations indicated poorer balance function, with > 60 s considered good, 30–60 s fair, and < 30 s poor [23]. 

### Dynamic balance ability

Was assessed using the Biodex Stability System (BSS; Biodex Medical Systems, Shirley, NY, USA), which features a 360° rotating platform with a maximum tilt of 20° [24]. Specialized software (Biodex, Version 1.32) used indices such as the Overall Stability Index (OSI), Anterior-Posterior Stability Index (APSI), and Medial-Lateral Stability Index (MLSI) for evaluation. APSI and MLSI represent platform displacements in the sagittal (Y) and frontal (X) planes, respectively, while OSI is their composite score (OSI = [(Σ(0– Y)² + Σ(0– X)² / samples)]^0.5).[25] Lower scores indicated better balance. Testing occurred in a quiet setting. Participants stood barefoot, one-legged, with eyes open, knees bent 15°, arms down, and feet on markers. After a 1-minute adaptive training and three practice trials to minimize anxiety and learning effects, the official test involved an 8-level stability test (most stable level) [26]. Each 20-cycle had automatic tilting. Three trials were conducted with a 10-second rest between them, and the average score was computed.

### Proactive balance ability

Was assessed using the Timed Up and Go Test (TUG) [27]. Participants sat in a chair with a backrest, seat height of approximately 45 cm, and armrest height of approximately 20 cm, leaning back against the chair with feet flat on the ground. Upon hearing “start,” participants rose from the chair, walked as quickly and safely as possible forward, crossed a marker line 3 m from the chair, turned around, and returned to sit back against the chair. The time from the “start” command to sitting back down was recorded. After one practice trial, two test trials were conducted, and the average of the two trials, rounded to two decimal places, was used in the analysis.

### Reactive balance ability

Was evaluated using the Push and Release Test (PRT), which assessed participants’ postural response to suddenly releasing an examiner’s hand placed on their back [28]. Participants stood in a comfortable position with their eyes open and pushed backward against the examiner’s palm. Upon sudden release, participants had to regain balance (by taking steps backward until they reached a stable position). The examiner ensured the participants’ safety during testing. For rating, the actual number of steps taken to regain balance (excluding steps to reposition feet) was measured (0 = 1 step, 1 = 2–3 small steps independently, 2 = ≥ 4 steps independently, 3 = steps with assistance, 4 = fall or inability to stand without assistance).

### Daily living balance ability

Was assessed using the Balance in Daily Living (BDL) scale recently developed by French scholars [29]. Participants were asked to complete seven daily household tasks requiring postural adaptation and multitasking. Each task was scored as follows: 1 = normal (no instability), 2 = adequate (patient uses safe strategies, anticipatory adaptation, no instability), 3 = abnormal (patient uses reflexive adaptation strategies, corrective adaptation, fall avoidance strategies, mild instability), and 4 = very abnormal (ineffective postural adaptation reflexes, severe instability). No score was assigned if a task could not be performed due to reasons other than balance impairment.

### Grip strength

Is assessed using a CAMRY^®^ electronic dynamometer. During the test, participants maintain neutral shoulder rotation and adduction, 90° elbow flexion, and neutral wrist and forearm positions. Participants perform three maximum-effort trials with verbal encouragement, with a 1-minute rest between each trial. The mean grip strength (HGS) is calculated as the average of the three measurements in kilograms.

### Manual dexterity

Is objectively evaluated using the Jebsen-Taylor Hand Function Test (JHFT), which measures hand function in daily living activities. JHFT comprises seven subtests: (1) writing a standard sentence, (2) turning over five cards, (3) picking up and replacing small objects, (4) simulating eating with a spoon and five beans, (5) stacking four raw beans on a board, (6) flipping five large empty cans, and (7) flipping five full cans. The completion time for each subtest is recorded to the nearest second and summed to obtain a total score. JHFT is a reliable and valid assessment tool with established psychometric properties in older adults and is widely used to assess hand dexterity in various contexts [30]. 

### Life-space mobility

Is evaluated using the Life-Space Assessment (LSA). This scale assigns scores based on the range of activities (1–5 points for bedroom, home, outside home, community, and beyond the community), frequency of activities (1.5–4.5 points for 1 week, 2–3/week, 4–6/week, and daily), and independence (1, 1.5, and 2 points for assistance, assistive devices, and no assistance, respectively). The LSA total score is calculated as the sum of area scores (area score = range × frequency × independence), ranging from 0 to 120 points, with higher scores indicating greater mobility [7]. 

### Secondary outcomes

#### Smart monitoring devices

Used in this study are the Electronic wristbands, which have a battery life of 30 days and are worn on the dominant hand to measure step count, heart rate, and blood oxygen saturation levels, providing an objective quantification of daily activity and cardiopulmonary function. Data from the wristband are collected via separate Gmail accounts for each participant and extracted into Excel documents using the mobile application. The parameters obtained are based on the 30-day averages of the aforementioned variables.

#### Exercise adherence

Is calculated as the percentage of personal activity participation times relative to the total number of interventions.

### Data collection and management

The data collection team consists of two medical graduate students and one community worker, primarily operating at rural community service centers. Data collectors must undergo standardized training and be fluent in local dialects. Table 2 shows the five measurement time points: baseline before randomization (T0), mid-intervention (T1), post-intervention (T2), and follow-up (T3 and T4). After data collection, participants’ identities will be anonymized using initial-number combinations, and handwritten records will be digitized. Two researchers will independently enter data into Excel. Software tools will detect inconsistencies and generate reports. After verification and cross-checking, data will be stored in an electronic database accessible only to principal investigators, data consultants, and data entry staff.

**Table 2:**
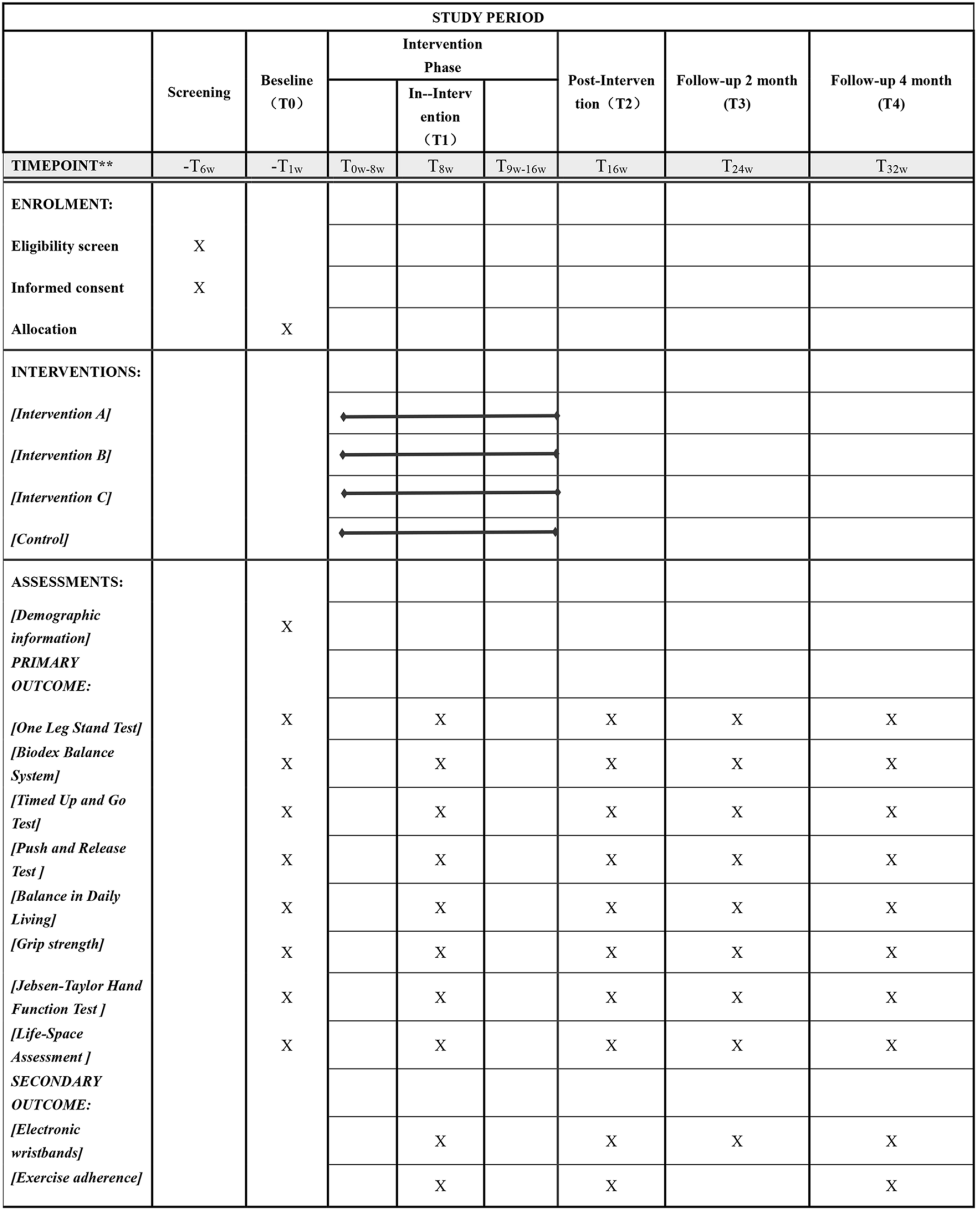
Study assessment points and timelines

### Data analysis

SPSS 26.0 software was used for statistical analysis, with a significance level set at *p* < 0.05. Initially, the Shapiro-Wilk test will be employed to assess the normality assumption for quantitative data. Based on these results, appropriate statistical methods will be selected to compare participants’ demographic and other baseline characteristics before the intervention. If the data are normally distributed, an independent-sample t-test will be utilized; otherwise, the Mann-Whitney *U* test will be applied. The primary outcome measures, continuous variables, were analyzed using generalized estimating equations (GEEs). In cases of significant interactions between group and time factors, a simple effects analysis will be conducted to elucidate the sources of the differences. If the simple effects analysis of time factors yields significant results, post hoc tests will be performed using the Sequential Bonferroni Procedure to identify specific time points of substantial differences. Secondary outcomes included descriptive statistics on exercise adherence after intervention.

## Discussion

To our knowledge, this study is designed as the first randomized controlled trial to investigate the potential effects of the multidisciplinary team-based M-mBHT program on rural older adults through four parallel groups with equal training doses over 16 weeks. Therefore, the design of this study protocol is expected to complement the existing gaps in balance and hand function training, potentially providing rural older adults with a low-cost and efficient exercise program.

Previous studies have shown that balance training is divided into static balance (i.e., static-continuous balance) and dynamic balance (i.e., dynamic-continuous, active, and reactive balance) exercises, comprising two-thirds of the total training time [31, 32]. Hand training, based on acupoint principles and constituting one-third of the time, uses massage and kneading to stimulate hand receptors, exciting the cerebral cortex [33], and boosting blood flow, flexibility, and strength. This provides a theoretical reference for the combination in the design of this research protocol, which is anticipated to contribute to improving the effectiveness of the integrated program for balance ability and hand function in rural older adults.

Interestingly, this study employs an innovative, comprehensive index system (multidimensional balance ability, hand function, life-space mobility, and intelligent monitoring device data) to evaluate the anticipated short-term and long-term effects of the M-mBHT program. Most outcome variables use objective assessment methods, which are expected to reduce measurement errors and risks associated with subjective evaluations. Additionally, we have constructed a complete assessment framework for balance ability (static, dynamic, proactive, reactive, and daily living balance), covering five dimensions. This framework is designed to comprehensively understand individual performance in balance and potentially identify focuses for subsequent improvements. A meta-analysis indicates that wearable technology interventions lasting more than 12 weeks can achieve more significant improvements in activity ability [34]. Electronic wristbands, as user-friendly and comfortable trackers, are intended to effectively monitor older adults’ daily activities and cardiopulmonary functions. The 16-week structured intervention across four multicenter groups, with outcomes evaluated at 2, 4, 6, and 8 months post-randomization, is designed to ensure ensures data continuity. This provides a time window to explore the effectiveness of the intervention programs at different stages and to investigate the impact of cycle factors on outcome indicators.

The M-mBHT program is comprehensive, incorporating sensory, progressive circulation, group supervision, and online integration. The decline in physical function and balance ability in older adults is often related to sensory weakness. Maintaining balance and engaging in exercise is theorized to require the coordinated action of sensory information such as vision, auditory, muscle tension, support surface, and vestibular sense in both static and dynamic states [26]. Considering the rural living environment, this study includes flat ground and safe, convenient sandy ground in balance training to change the contact surface. Visual and auditory deprivation and certain disturbances are also introduced. Repeated and intermittent visual occlusion **is** hypothesized to enhance balance training by potentially increasing cortical phase synchronization and improving information transmission efficiency and reaction speed [35]. Intermittent auditory deprivation aims to simulates real-life challenges, potentially increasing reliance on vision and proprioception, and thus may enhance muscle strength and balance outcomes. This approach is designed to be simple, with low venue requirements and minimal skill demands on seniors, aiming to ensure wide adaptability.

Considering the learning capabilities of older adult individuals, we established four WeChat groups to manage four groups. Teaching videos were created and uploaded to the WeChat public account for dissemination within the group chat, along with online support for addressing any issues encountered by participants. A review analyzing exercise compliance among older adults indicates that supervised training schemes generally exhibit higher adherence rates [36]. Participants who failed to attend on time were contacted via WeChat to enhance their motivation and ensure the timely completion of the program. Recognizing the impact of social isolation on function [37], the intervention aims to integrates person-environment fit, using local rural community centers to form neighborhood exercise groups, which may enhance social interaction. Delivering potentially effective exercises for rural seniors is complex and is expected to demands teamwork among diverse healthcare specialists. Experts in medicine, psychology, and sociology develop and review our programs. To enhance community medical services, the Chinese government has established medical alliances in recent years [38]. Given this context and the study hypothesis, combining institutional and community healthcare workers represents a proposed model for program implementation, anticipated to be effective based on available frameworks.

The proposed M-mBHT program has several limitations that may influence the generalizability of findings. First, due to the limited number of research sites, the sample may not be fully representative of China’s rural older adults. Second, the absence of muscle mass assessment could be a limiting factor, as the 16-week intervention period might not be sufficient to observe significant increases in muscle mass among older adult participants. Additionally, because of the nature of the intervention, participants were not blinded, which may be a potential limitation in maintaining objectivity in the assessment of self-reported outcomes. Finally, effectively integrating rural community resources under national rural policy guidelines to provide scientifically sound and feasible exercise programs for older adults remains a unique challenge. Future multi-center, large-scale randomized controlled trials (RCTs) are necessary to further validate the research findings regarding the impact of the M-mBHT program on the health management of rural older adults.

In the context of healthy aging and changing socio-demographic changes, maintaining the multidimensional balance ability (static, dynamic, proactive, reactive, and daily living balance), hand function (grip strength and manual dexterity), and life-space mobility of vulnerable groups such as rural older adults is essential for promoting health equity and achieving healthy aging. Thus, a high-quality research protocol design is expected to be crucial for establishing scientific principles and potentially improving the activity levels and balance ability and functional status of rural residents. If the hypothesized benefits are observed, the findings may contribute to evidence-based strategies for enhancing functional health in rural older adults. However, definitive conclusions await the trial’s completion and data analysis.

## Electronic supplementary material

Below is the link to the electronic supplementary material.

Supplementary Material 1

Supplementary Material 2

## Data Availability

Data sharing is not applicable to this article as no datasets were generated or analysed during the current study. However, de-identified study data will be provided upon reasonable request to the corresponding author following completion of the trial and peer review. Data may be stored in a public repository in accordance with the journal’s open science policies.
